# Evaluating Signalization and Channelization Selections at Intersections Based on an Entropy Method

**DOI:** 10.3390/e21080808

**Published:** 2019-08-18

**Authors:** Yang Shao, Xueyan Han, Huan Wu, Christian G. Claudel

**Affiliations:** 1 Traffic and Road Engineering Center, Highway Academy, Chang’an University, Xi’an 710064, China; 2 Human Resource Department, Xi’an Shiyou University, Xi’an 710065, China; 3 Cockrell School of Engineering, University of Texas at Austin, Austin, TX 78712, USA

**Keywords:** entropy evaluation method, traffic conflict, VISSIM, vehicle emission, restriction policy

## Abstract

Direct left turns (DLTs) could cause traffic slowdown, delay, stops, and even accidents on intersections, especially on no-median roads. Channelization and signalization can significantly diminish negative impact of DLTs. In China, a total of 56 large and medium-sized cities, including 17 provincial capitals, have adopted vehicle restriction policies due to traffic congestion, vehicle energy conservation and emission reduction, which cause travel inconvenience for citizens. This paper mainly studies signalization and channelization selections at intersections based on an entropy method. Based on the commonly used three evaluation indexes, the number of vehicles, CO emissions and fuel consumption have been added. The entropy evaluation method (EEM) method is innovatively used to objectively calculate the weight of the six indexes, which carry out the optimal traffic volume combinations for intersections of present situation, channelization and signalization. A VISSIM simulation is also used to evaluate the operating status of three conditions. The results show that EEM could help enormously in choosing different methods at a certain intersection. With the EEM, six indexes decrease by 20–70% at most.

## 1. Introduction

Left-turning maneuvers are considered one of the most hazardous traffic maneuvers, since turning vehicles have to cross in front of the oncoming through traffic [1,2]. Left-turn design at intersections in urban areas has long been considered a dilemma. Providing left signal control for left-turning vehicles increases the delay [3], while not doing so increases conflicts between the left-turning vehicles and the through traffic in the opposite direction [4,5]. To eliminate this problem, many alternative solutions have been proposed to improve the performance of intersections with left-turning vehicles. Examples include signal timing estimation [6,7], exclusive left lane design [8] and state-of-the-art technology like pilotless automobiles [9]. Among all these solutions, facility design remains an important approach to existing problems.

During the past few decades, various left-turn designs have been used on urban intersections to reduce problems accompanying direct left-turn (DLT) vehicles. The difficulty of completing this movement is evident in crash statistics, indicating that 45% of all crashes that occur at intersections throughout the United States involve left-turning vehicles, even though left-turning movements represent a disproportionately small percentage (10–15%) of all approaching traffic [10,11]. DLT vehicle movements from arterial streets or collector roads are prohibited by using non-crossing median and/or directional median openings. Left-turning vehicles will be guided to detour downstream to U-turn locations instead of a left turn. Superstreet intersections, crossover displaced left turn intersections, and upstream signalized crossover schemes are common median U-turn intersection designs [12,13,14]. Left-turn vehicles also cause pedestrian-vehicle conflicts [15]. Previous research and studies of U-turn designs have proven they have an exclusive role in reducing travel time, delay, and traffic conflicts and in improving safety at intersection areas [16,17,18]. The only restriction of U-turns is the requirement for a large median width, which limits its application.

Channelization, an exclusive left-turn lane, is popularly implemented for left-turn problems. The set of left-turn lanes depends on the ratio of left-turn vehicles [19,20]. Displaced left-turn intersections that resolve the conflict between left-turn and opposing-through movements at the pre-signal are probably the most extensively used innovative intersection designs [21]. Left-turn lanes appear to contribute to crashes without accounting for endogeneity [22], and a left-turn waiting area could increase the capacity at intersections [23].

Signalization is another popular way to reduce junction traffic conflict. A separate turn phase is often used on the approach leg to an intersections with heavy left turns [24,25]. The most common identified guidance for protected left-turn phases is to use a threshold based on the cross-product of left-turn volume and opposing through movement [26]. A left-turn lane with signal control could improve the operational performance of left-turn movement at signalized intersections in China [27,28]. Stop-controlled intersections have a strong association between high crash risk and high traffic speed, especially for older female drivers [29].

Some cutting-edge technologies also pay attention to left-turn problems. Augmented reality (AR) technology can offer a very realistic environment for enhancing driver reaction to different road design and traffic operations scenarios [30]. The optic technique uses an optical combiner for combining real and virtual objects, and the video technique uses a computer or a video mixer to combine the video of the real world, from video cameras, with virtual images (computer-generated) [31]. Head-up display (HUD) devices that are gradually being popularized in automobiles have helped drivers better process traffic information and have reduced traffic accidents in recent years [32,33]. Head-mounted display (HMD) devices, e.g., Microsoft Hololens, is another tool with huge potential for intelligent transportation technology for reducing traffic problems in the future [34,35].

In addition to turning conflicts, some researchers have also studied the relationship between vehicle emissions and traffic delay in urban areas [36,37]. In China, a total of 56 large and medium-sized cities, including 17 provincial capitals, have adopted vehicle restriction policies due to traffic congestion, vehicle energy conservation and emission reduction, which covers the majority of developed cities in China. In the meantime, the policies are various in restrict scopes and guidelines as in Table 1, which not only causes inconvenience to local residents, but also causes great trouble to vehicles from other cities [38]. Traffic delay and emissions have a strong connection, which means that reducing delay could reduce emissions [39,40,41]. Different route selections and stop strategies could reduce bus emissions [42]. Vehicle gap changes could also affect fuel and emission performance [43].

As China’s urbanization continues, vehicle ownership, including the above-mentioned restricted cities, is still growing. In addition, traffic congestion and vehicle exhaust pollution may still intensify [44]. The restriction scope is larger and larger and more cities are joining in the vehicle restriction policy movement while the restriction policies have been proved to be ineffective for diminishing traffic problems and congestion [45]. It is necessary to tap the potential of existing road traffic facilities and traffic management as much as possible and try to use technical methods instead of administrative polices. While numerous guidelines for the selection of left-turn phasing have been developed, there is no widely recognized guideline or criterion for the use of left-turn phasing under specific traffic conditions [46]. In this study, an exclusive left-turn lane (ELTL) design for no-signal intersections on the way without a median is proposed to diminish left-turn conflict and delay. To reach that goal, two strategies are used. The first is channelization, an exclusive left-turn lane with stop sign control, named plan 2 in the following. The second is signalization, an exclusive left-turn lane with signal light control, named plan 3 in the following. The present situation, without an exclusive left-turn lane, named plan 1, was also evaluated for comparison. It could be easily predicted that both methods could reduce delay and emissions, but how to select a proper method for a certain intersection remains a problem.

**Figure 1:**
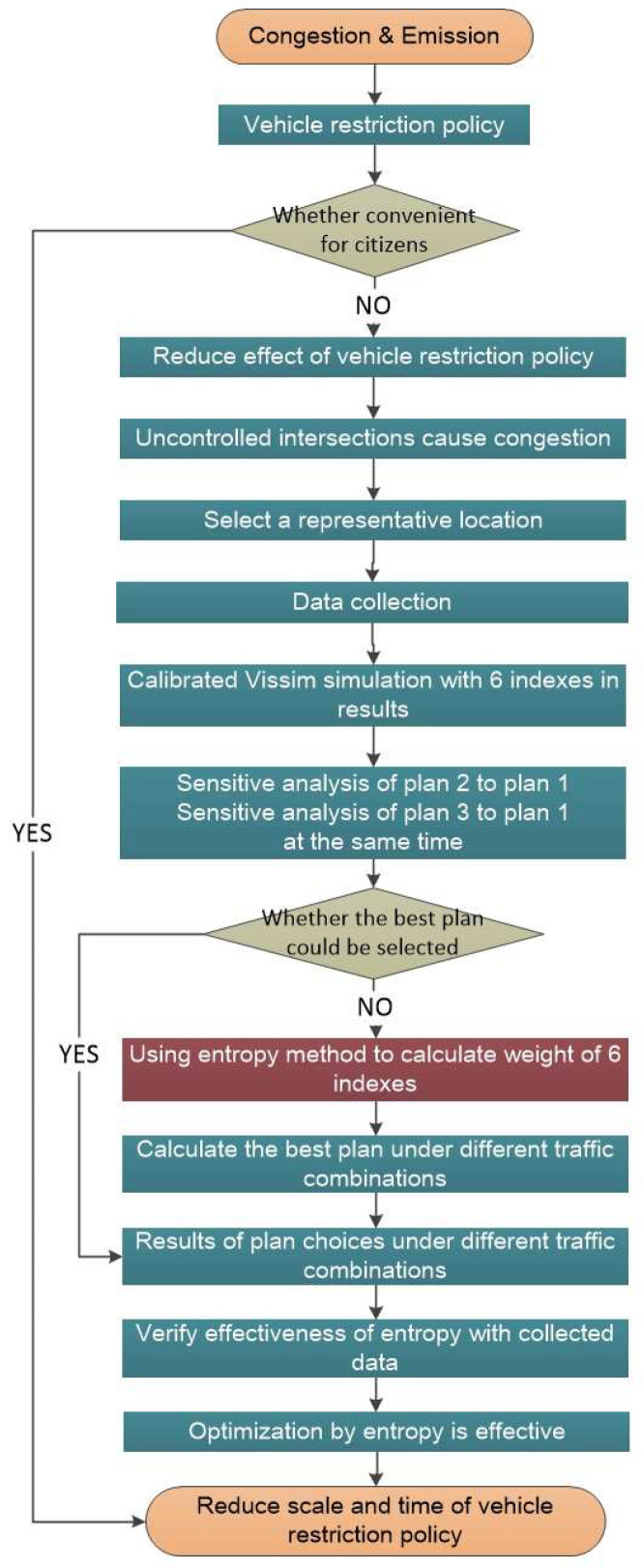
Flowchart of selecting stop and signal control at intersections with entropy evaluation method.

The primary objective of this study is evaluation of the entropy evaluation method (EEM) based on selection of the three situations, for which it is very easy to determine the priority level and reconstruction method for large scale intersections, and making the traffic flow more smoothly to achieve vehicle energy conservation and emissions reduction, and to use the restriction policy instead. The travel time, delay, number of stops, number of vehicles, CO emissions, and fuel consumption are evaluated for various traffic situations. The EEM is widely used in evaluating and calculating plans of multiple elements, such as physics [47], information [48], medical science [49,50], business [51], environment [52], statistics [53], finance [54] and other interdisciplinary subjects [55,56]. This is the first time to introduce EEM into transportation evaluation, which could judge the plans synthetically instead of several indexes separately. The rest of this article is organized as follows: Section 2 contains a problem statement and Section 3 details the design schemes. Section 3 includes data collection, VISSIM simulation calibration, and sensitivity analysis. In Section 4, we discuss the EEM method calculation process and verify the validity of the EEM. Conclusions are drawn in Section 5, see Figure 1.

## 2. Problem Statement and Design Schemes

### 2.1. Problem Statement

Take Xi’an as an example: the number of vehicles in Xi’an is 3 million and ranked 8th in China at the end of May, 2018. The restriction policy as Table 1 shows is appropriate for both local and nonlocal vehicles on weekdays. Ideally, 20% vehicles are restricted on one single weekday, which means 600,000 vehicles. However, vehicles had a 300,000 annual growth in the past two years according to the Department of Xi’an Vehicles [57]. That is to say, after the implementation of the restriction policy 12 years from 2018, the congestion and pollution situation would recover to the level in 2018. In addition, there was a total increase of 910,000 people in the past two years in Xi’an, and the second vehicle purchase would result in a high probability of vehicles increasing in Xi’an [58].

**Figure 2:**
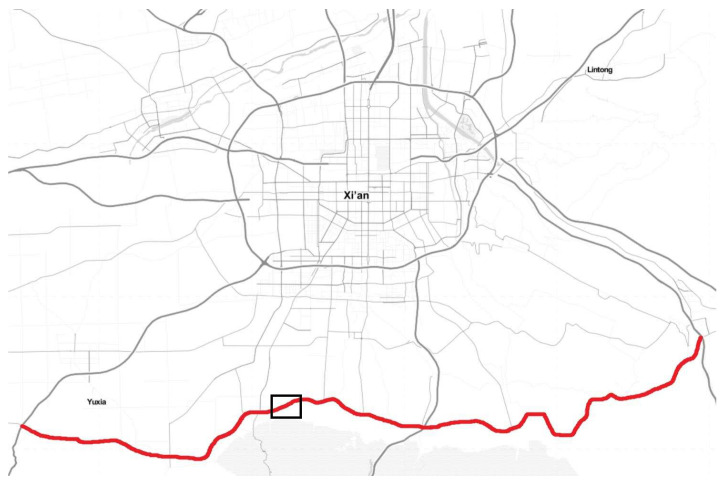
Investigated road S107 scheme. The investigation segment length is 84.3 km and the black box is the T-intersection location discussed.

Channelization and control method (yield control or signal control) are the most popular schemes used to solve traffic problems for non-controlled T-intersections. Left-turn vehicles may cause delay, stops, and even safety hazards in non-controlled T-intersections. It is not easy to judge which method should be used and which non-controlled T-intersection has the priority when too many intersections exist. In one part of China’s Xi’an district loop road S107, 20 signalized intersections and 87 non-controlled T-intersections were distributed in 84.3 km according to our investigation; see Figure 2. Choosing which intersections to channelize and which intersections to add traffic light control to becomes significant. This paper will focus on a comparison of three solutions and the computation method of how to select the proper reconstruction plan.

### 2.2. Design Scheme Description

To distinguish non-controlled situations, channelization, and signal control, we use plans 1, 2, and 3 to represent each respective plan. Plan 1 is the present situation without any control measures and serves as a benchmark for comparison. Plan 2 added ELTL and stop/yield signs as control methods compared to plan 1. Plan 3 added traffic lights instead of stop/yield signs compared to plan 2.

**Figure 3:**
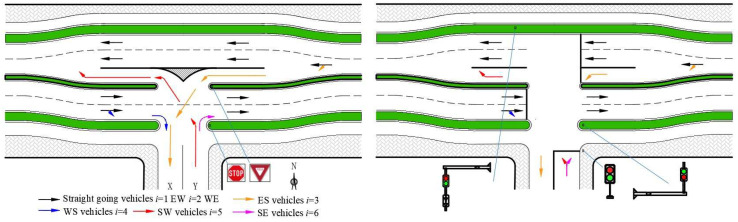
Illustration of channelization plan 2 and signalization plan 3.

**Reconstruction plan 2, channelization.** An illustration of the general design of plans 2 and 3 for an arterial-collector street intersection is shown in Figure 3.

In Figure 3, WENS indicates the four cardinal directions, X means the entry lane of the collector street and Y means the exit lane of collector street. Six traffic flows are shown in Figure 3 using different colored arrows. The flows i=1 and i=2 are straight-going vehicles of the East to West (EW) and West to East (WE) lanes, respectively. These two flows are the main traffic flows with a relatively high traffic volume. The flows i=1 and i=2 always have a high speed, and they force turning vehicles (flows i=5 and i=6) to wait to merge. Flows i=4 and i=6 are right-turning vehicles, one from west to south and one from south to east. They do not influence the others much. Flow i=3 represents the turning vehicles from east to south; if the opposite flow is high, they need to yield to flow i=2 and wait in the middle lane and influence flow i=1 (flow i=1 will change lanes, or stop to wait). This will cause delays and safety hazards for flow i=1. Flow i=5 represents vehicles moving from south to west. Flow i=5 is the most complicated because flow i=5 must yield to the flows i=2 and i=1 to find an acceptable headway gap, meaning flow i=5 may need to wait for a very long time if there is no median and the traffic volume is high. Each car of flow i=5 must obey stop signs when they reach stop lines before they turn.

The middle lane, ELTL, in Figure 3 is a lane for turning vehicles to decelerate, wait, accelerate, and merge. Both sides of the road move outward slightly by a half-lane width. Separating the turning flow and straight flow is the core of this design, which reduces traffic conflicts, traffic delay, and number of stops. For flow i=3, vehicles can decelerate and wait without influencing flow i=1. For flow i=5, the waiting time for a headway gap now only depends on flow i=2. Acceleration and merging can be done at the ELTL. Straight-going vehicles, flow i=1, will avoid deceleration, sudden stops, and safety hazards.

**Reconstruction plan 3, channelization and signalization.** As Figure 3b shows, traffic lights instead of stop/yield signs are used to control different traffic flows. Synchro 7 was used to compute optimal timing cycles under different traffic volume combinations. The traffic volume combinations are introduced amply in Section 3, sensitivity analysis of operational performance. Traffic operation situations could be done with Synchro 7 optimal timing cycles input into VISSIM. A final plan can be computed with comparison among these three plans.

**Figure 4:**
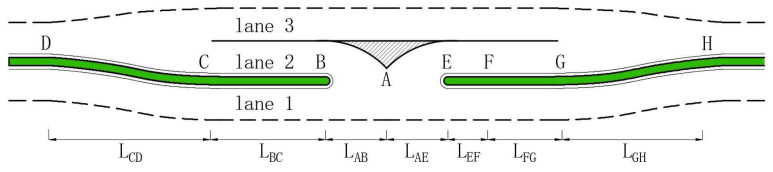
Illustration of ELTL design.

**Geometry** The ELTL design’s geometric dimensions for a highway speed of 80 km/h are shown in Figure 4. Lanes 1 and 3 are the inner straight-through lanes of WE and EW, respectively. Lane 2 is the ELTL for flows i=3,5 to decelerate and accelerate. All lengths are based on AASHTO ‘Highway Capacity Manual’ [59], ‘A policy on geometric design of highways and streets’ [60], and on previous studies [61,62]. All parameters are described in Table 2.

All different section lengths are input into the simulation model to evaluate the ELTL performance for the T-intersection with a design speed of 80 km/h.

**Development of Simulation Model.** Ideally, data should be collected before and after road improvement (e.g., before and after DLT and ELTL application) for comparative analysis. This is difficult to do in practice, especially when the ELTL is not actually built. Therefore, it is necessary to use traffic simulation software to evaluate the changes of traffic operating state before and after the improvement. The VISSIM simulation software has been applied in the field of traffic simulation for more than 40 years [63]. Some achievements have been made in the research on some key parameters in traffic flow [64], including the car following model [65,66], driving behavior model [39], lane change model [67] and U-turn model [68]. The accuracy of VISSIM in traffic flow simulation is widely recognized [18,69]. Therefore, in this study, the simulation comparison of DLT and ELTL, as well as the sensitivity analysis of the operational measures of ELTL, were simulated by VISSIM.

To guarantee the accuracy of the simulation, the geometric road parameters input into VISSIM must be emphasized. In addition to the geometric length of each section of ELTL mentioned above, the map of the actual intersection is taken as the base map, Autodesk CAD software is used to draw the road, and both are imported into VISSIM to guarantee the accuracy of the geometric size and trend of the road in the simulation process consistent with the actual situation. In this study, turning vehicles must yield to straight-going vehicles. Turning vehicles must wait for an acceptable headway gap to cross the street.

## 3. Data Collection and VISSIM Simulation

### 3.1. Data Collection

Realistic traffic data are needed for VISSIM model calibration. In addition, 32% of traffic accidents and 34% of fatal traffic accidents happen on roads without a median according to statistics in China [70]. A typical location of left turns without a median or traffic light on road S107 was selected for the investigation, which has high operating speed, large traffic volume, many left-turning vehicles, a lack of signals, a far distance from upstream and downstream signalized intersections, good sight distance, and not too many bicycles and pedestrians, which are the requirements for field data collection.

**Figure 5:**
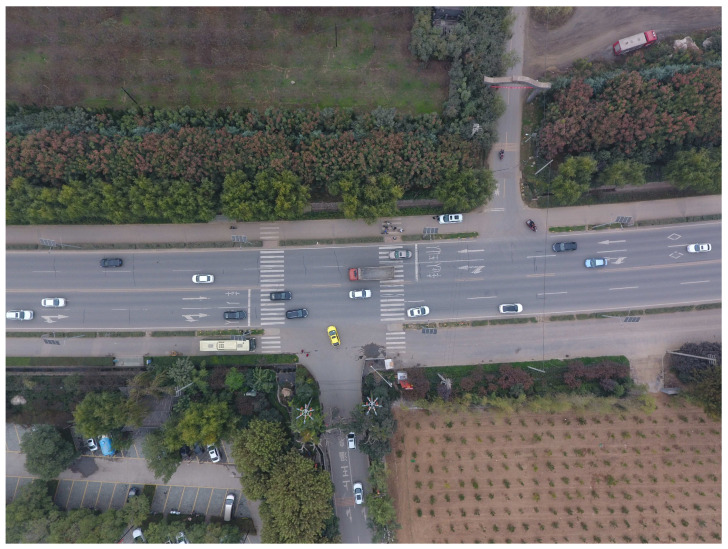
T-intersection at Shangwang Village Resort. Coordinates:108.845118,34.057511. Present intersection image taken by a drone at a 130-m height.

A T-intersection with a two-lane-wide collector street is the only entrance and exit of Shangwang Village Resort, and the arterial street is the four-lane s107 provincial trunk highway with a speed limit of 80 km/h. No median strip exists, but there is a 1.2-m-wide greenbelt dividing the 3-m-wide non-motor lane from the main lanes. The Shangwang Resort receives more than 2 million tourists annually and its revenue is nearly 100 million yuan (14 million dollars). Many tourists are attracted to the resort, and the majority of them drive cars, which means the turning vehicles (TVs) are mostly cars (flows i=3,4,5,6 were 100% cars during the investigation). This T-intersection is 1.3 km from the next signalized intersection to the west and 3.4 km from the next signalized intersection to the east, which means the vehicle operating speed in this section is high if the vehicle does not want to turn into the resort. Owing to the large traffic volume, TVs always need to wait a long time to enter or leave. However, straight-going vehicles (SVs, flows i=1,2) must slow down or even stop when TVs cannot turn quickly (Figure 5).

**Figure 6:**
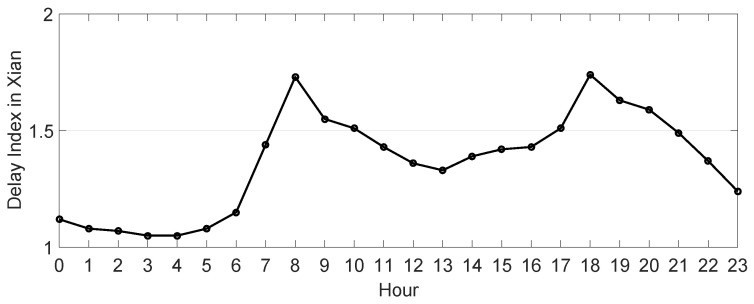
The 24-h congestion delay index for Xi’an on 2018.10.13 [71,72]. The peak appeared between 8:00 a.m.–9:00 and 6:00 p.m.–7:00 p.m., and the valley, excluding late night, appeared from 1:00 p.m.–2:00 p.m.

Two video cameras were used for the vehicle amount count and two radars used in two directions to collect trajectories and speed of vehicles. Field data collection was conducted during weekday and weekend peak and valley periods. Data were collected during a good weather day without traffic congestion, accidents, or roadway maintenance. In total, the research team recorded 6 h of traffic data in the field. According to the *2017 Traffic Analysis Reports for Major Cities in China* [71] proposed by AutoNavi Traffic big data, the morning peak appeared from 7:00 a.m.–9:00 a.m., the evening peak appeared from 5:00 p.m.–7:00 p.m., and the valley, excluding late night, appeared from 12:00 p.m.–2:00 p.m. [20]. In addition, a 24-h congestion index in Xi’an on 20181013 was in Figure 6 [72].

The collected data are as follows:

- Collect all vehicle speeds and types in each lane during collection period.
- Record every turning vehicle and type.
- Collect all vehicle trajectories.

The six hours of collected data on Friday and Saturday are listed in Table 3:

The highest volume appeared on Saturday evening and this data was used in the following part as listed in Table 4.

The collected data show the following characteristics:

1. The majority of running speeds for flows i=1,2 were much lower than the speed limit and design speed (80 km/h).
2. Flow i=2 was strongly influenced by TVs (flows i=3,5).
3. Flow i=1 was influenced by TVs (flows i=3,5) but not as strongly as flow i=2.

### 3.2. Calibration of VISSIM Simulation Model

The VISSIM parameters need calibration and validation with the investigation data to ensure simulation accuracy. Traffic data, capacity, and geometric measures were collected to calibrate the simulation model in VISSIM.

Before the VISSIM simulation model can be used for modeling the capacity of all six flows, the model must be calibrated and validated against field data to ensure that VISSIM provides reasonable capacity estimates for all movements [73]. Simulation model calibration in this stage followed the normal calibration procedures proposed by previous studies [74,75,76]. The data collected in the Shangwang Resort were considered the inputs in VISSIM:

1. The percentages of large vehicles and cars are 4% and 96% in EW and 8% and 92% in WE, respectively.
2. From west to east, the flow i=4 ratio is 8% and the flow i=2 ratio is 92%.
3. From east to west, the flow i=3 ratio is 4% and the flow i=1 ratio is 96%.
4. The flow i=5 ratio is 54% and flow i=6 ratio is 46% on the collector street.
5. The headway of vehicles ranges from 1.5 to 15.1 s with an average of 6.9 s.
6. Turning speed ranges from 0 to 28.5 km/h.
7. The expectation speed is 25.2–81.4 km/h in EW and 0–91.4 km/h in WE.

Several calibrated parameters are available in the VISSIM simulation model: the gap-accepting model, car-following model, and lane-changing model. Capacity is mainly used for route choice in road network calibration. This index can reflect multiple properties of the model, and is very sensitive to route choice behavior. An accurate capacity is required for the simulation. The capacity can be calculated by Equation (1):

(1) $$C=\frac{3600}{\bar{h_{t}}},$$

where *C* denotes the ideal capacity (veh/h) and $\bar{h_{t}}$ the average minimum headway (s). The capacity used to estimate simulation error is the mean absolute percent error (MAPE), which can be calculated by Equation (2):

(2) $$MAPE=\frac{1}{n}\sum_{i=1}^{n}\left|\frac{C_{v}^{i}-C_{f}^{i}}{C_{f}^{i}}\right|,$$

where *n* denotes the six different flows, $C_{v}^{i}$ is the capacity simulated in the VISSIM model (veh/h), and $C_{f}^{i}$ is the investigated capacity (veh/h). The calculated MAPEs are displayed in Table 5:

The capacity error between the VISSIM simulation model and reality is 4.3%. Model validation results suggested that the calibrated VISSIM simulation models provided reasonable capacity estimates that can be considered acceptable in practical engineering applications [77,78].

### 3.3. Vissim Calculation of Operational Measures

Travel time, delay, and number of stops are the most commonly used indexes to evaluate the effectiveness of comparison between different operational measurements [79,80]. In recent years, China has not only faced traffic congestion problems, but also air pollution problems [81]. Many cities have adopted private vehicle restrictions to alleviate traffic congestion and urban air pollution [38]. Several indexes of the simulation model can be obtained from VISSIM, such as CO emissions [quantity of carbon monoxide (grams)], NO${}_{x}$ [quantity of nitrogen oxides (grams)], VOC [quantity of volatile organic compounds (grams)], number of vehicles, and fuel consumption (U.S. liquid gallons) [69]. CO, NO${}_{x}$, VOC, number of vehicles, and fuel consumption exhibit the same change trend according to results of the VISSIM simulation. Considering that the main purpose of this research is the improvement of traffic conditions and that of air quality is auxiliary, six indexes are considered and calculated to evaluate operational features of the three aforementioned plans, including travel time, delay, number of stops, number of vehicles, CO emissions, and fuel consumption.

**Travel time.** Travel time means the average time it takes for all vehicles to pass a given distance. In the VISSIM simulation, “it consists of a from section and a to section. The mean travel time from traversing the from section up to traversing the to section, including the waiting time and/or holding time, is calculated as well as the distance traveled between the start section and destination section” [69]. It can be calculated using Equation (3):

(3) $$T_{{}_{i}}^{k}=\frac{\sum_{j=1}^{Q_{i}^{k}}t_{{}_{ij}}^{k}}{Q_{i}^{k}}\;\forall i=1,2,3,4,5,6\;\forall k=1,2,3,$$

where $T_{{}_{i}}$ denotes the travel time of each traffic flow in Figure 3 under plans 1, 2, or 3; $t_{{}_{ij}}$ denotes one single vehicle’s travel time in each flow; and $Q_{i}$ denotes the total amount of passed vehicles of each flow with the corresponding design plan. k=1,2,3 denotes different plan numbers.

**Delay.** Delay means the difference between actual travel time and the driver’s expected travel time. The reasons for the difference include traffic interference, traffic management, and control measures. In this study, delay includes stop delay and travel delay:

(4) $$D_{i}^{k}=d_{i1}^{k}+d_{i2}^{k}\;\forall i=1,2,3,4,5,6\;\forall k=1,2,3,$$

where $D_{i}$ denotes the total delay of each flow in Figure 3 under plans 1, 2, or 3; $d_{i1}$ denotes its stop delay of each flow; and $d_{i2}$ denotes its travel delay of each flow. k=1,2,3 denotes different plan numbers.

**Number of stops.** In the VISSIM simulation, this measure indicates all situations in which a vehicle comes to a standstill (speed = 0), except stops at public transport stops and in parking lots [69]. It can be calculated by the total stop numbers of each flow divided by the total amount of vehicles in this flow:

(5) $$S_{{}_{i}}^{k}=\frac{\sum_{j=1}^{Q_{i}^{k}}s_{{}_{ij}}^{k}}{Q_{i}^{k}}\;\forall i=1,2,3,4,5,6\;\forall k=1,2,3,$$

where $S_{{}_{i}}^{k}$ denotes the average number of stops of each flow in Figure 3 under plans 1, 2, or 3; $s_{{}_{ij}}$ denotes the number of stops of each vehicle; and $Q_{i}$ denotes the total number of vehicles in this flow for each corresponding plan. k=1,2,3 denotes different plan numbers.

**Number of vehicles.** A node was set in the VISSIM simulation model and all flows’ vehicle numbers were collected. Number of vehicles could reflect the traffic capacity and could be used to compare the differences between the three plans. At least the number of vehicles was unchanged or increased under different plans’ operating conditions. It can be calculated as the total number of vehicles of all flows:

(6) $$NV_{i}^{k}=\sum_{j=1}^{6}nv_{i}^{k}\;\forall i=1,2,3,4,5,6\;\forall k=1,2,3,$$

where $NV_{i}^{k}$ denotes the total number of vehicles of each flow in Figure 3 under plans 1, 2, or 3. k=1,2,3 denotes different plan numbers.

**CO emissions.** Node evaluation also determines exhaust emissions. The basis for these are formed by standard formulas for consumption values of vehicles from TRANSYT 7-F, a program for optimizing signal times, as well as data on emissions from Oak Ridge National Laboratory, U.S. Department of Energy. The data refer to a typical North American vehicle fleet and does not differentiate between individual vehicle types. Thus, node evaluation is used to compare the emissions of different scenarios [69]. CO emissions can be calculated as

(7) $$CO_{{}_{i}}^{k}=\frac{\sum_{j=1}^{Q_{i}^{k}}CO_{{}_{ij}}^{k}}{Q_{i}^{k}}\;\forall i=1,2,3,4,5,6\;\forall k=1,2,3,$$

where $CO_{{}_{i}}^{k}$ denotes total CO emissions of each flow in Figure 3 under plans 1, 2, or 3; $CO_{ij}$ denotes CO emissions of each vehicle; and $Q_{i}$ denotes the total number of vehicles in this flow for each corresponding plan. k=1,2,3 denotes different plan numbers.

**Fuel consumption.** As with CO emissions statistics, fuel consumption can be obtained from VISSIM node simulation to compare the emissions of different scenarios. Fuel consumption can be calculated as

(8) $$F_{{}_{i}}^{k}=\frac{\sum_{j=1}^{Q_{i}^{k}}f_{{}_{ij}}^{k}}{Q_{i}^{k}}\;\forall i=1,2,3,4,5,6\;\forall k=1,2,3,$$

where $F_{{}_{i}}^{k}$ denotes total fuel consumption of each flow in Figure 3 under plans 1, 2, or 3; $f_{ij}$ denotes fuel consumption of each vehicle; and $Q_{i}$ denotes the total number of vehicles in this flow for each corresponding plan. k=1,2,3 denotes different plan numbers.

### 3.4. Sensitivity Analysis of Operational Performance

The collected data cannot cover all possible traffic situations, which restricts the simulation and evaluation of the operational effects of the new design in this article. Different traffic situations were specified in VISSIM to further investigate and evaluate the three plans. The ratio of each flow is steady in the sensitivity analysis, while the traffic volume changes in all six flows. The results of this subsection could reflect the improvement degree of plan 2 to 1 and plan 3 to 1.

The sensitivity analysis comprises travel time, delay, number of stops, number of vehicles, CO emissions, and fuel consumption. The service volume 3430 veh/h is the corresponding volume when the design speed is 80 km/h with two lanes under service level E according to [59]. The arterial street volume ranges from 0.2–1.0 service volume, which is 686–3430 veh/h. The collector street volume ranges from 100–500 veh/h.

**Figure 7:**
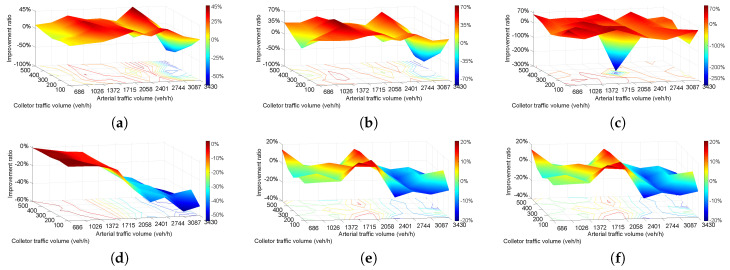
Improvement ratio of plan 2 compared to plan 1. (**a**) travel time; (**b**) delay; (**c**) number of stops; (**d**) vehicle number; (**e**) CO emissions; (**f**) fuel consumption.

Figure 7 shows the result for the improvement ratio of plan 2 to plan 1. The value of Figure 7 is calculated by Ratio = (plan2 − plan1)/plan1*100%. Figure 7a shows the travel time of 45 combinations (nine arterial street volumes and five collector street volumes). The majority of travel times improved significantly from 0–45% with plan 2 before 2744 veh/h. Travel time increased by more than 50% when arterial volume was 3087 veh/h. Regarding the performance of delay represented in Figure 7b, delay has a similar change trend with travel time on volume matrix. Delay reduced with arterial volume increased from 686–2744 veh/h, but the peak value 70% appeared at 1372-veh/h arterial volume and 300-veh/h collector volume. Delay increased when arterial volume exceeded 2744 veh/h, which is the same as travel time.

The number of stops reduced significantly, in general, 0–70%, except when arterial volume was 2058 veh/h and collector volume 300 veh/h, and the number of stops increased by more than 250%, as Figure 7c shows.

Based on the number of vehicles shown in Figure 7d, the entire range of results showed that plan 2 has a worse performance than plan 1. The results were not reduced too much when arterial volume was under 1372 veh/h and then reduced dramatically to 50% with increasing arterial volume. Figure 7d indicates that neither a separated left-turn lane or traffic light could increase traffic capacity under this condition. CO emissions (Figure 7e) and fuel consumption (Figure 7f) exhibit the exact same change, and the improvement ratio changed from 0–20% randomly and reached a peak value when arterial volume was 2058 veh/h and then reduced sharply to −20% with increasing arterial volume. Figure 7e,f indicate that plan 2 could reduce emissions and fuel consumption when traffic volume was under 2058 veh/h, after which plan 2 is incapable of having those effects.

Generally, Figure 7a–c represent that plan 2 has better performance than plan 1 when arterial street volumes were under 2744 veh/h. Figure 7e,f show that plan 2 is better when arterial volumes were under 2058 veh/h. Plan 2 could not improve the operating situation when arterial volumes were larger than 2744 veh/h and the collector street volumes cannot cause obvious differences among all six figures.

**Figure 8:**
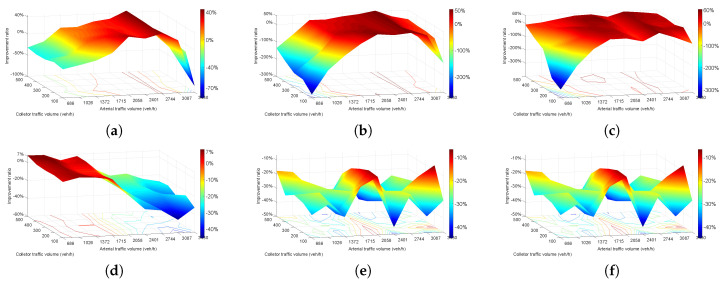
Improvement ratio of plan 3 compared to plan 1. (**a**) travel time; (**b**) delay; (**c**) number of stops; (**d**) vehicle number; (**e**) CO emissions; (**f**) fuel consumption.

Figure 8 shows the result for the improvement ratio of plan 3 to plan 1. The value of Figure 8 was calculated by Ratio = (plan3 − plan1)/plan1*100%. Figure 8a represents the change of travel time from plan 3 to plan 1. The travel time increased when arterial volumes were under 1372 veh/h and then started to decrease with increasing volume and reached a peak value of more than 40% when arterial volumes were 2744 veh/h. After 2744 veh/h, the result looks the same as Figure 7a and plan 3 could not improve the operating situation when arterial volumes were greater than 2744 veh/h. Delay of plan 3 to plan 1 is shown in Figure 8b, and the delay increased significantly to 300% when arterial volumes were under 1372 veh/h. Delay reduced from 0–50% when the arterial volumes ranged from 1372–3087 veh/h, and increased again to 100% at arterial volumes of 3430 veh/h. Figure 8c shows the number of stops of plan 3. Number of stops was reduced obviously among the majority of situations from 0–60% and only increased on a small scale when arterial volumes were under 1372 veh/h and collector volumes were under 300 veh/h.

Number of vehicles increased by 7% at most when arterial volumes were under 1715 veh/h and then gradually reduced to 40% finally in Figure 8d. Figure 8e,f indicate CO emissions and fuel consumption and they exhibit the same 100% trend. The two indexes both increased over the entire range and fluctuated within 10–40%.

## 4. Results

Under every arterial and collector volume combination, every index result in the VISSIM simulation was separately with six flows individually. In addition, the six flow results are taken together to obtain the node result corresponding to the volume combination. The calculation is as follows:

(9) $$X_{11}=\sum_{i=1}^{45}X_{1}\left(i\right),\;X_{22}=\sum_{i=1}^{45}X_{2}\left(i\right),\;X_{33}=\sum_{i=1}^{45}X_{3}\left(i\right),\;$$

where *X* denotes the following six indexes: travel time, delay, number of stops, number of vehicles, CO emissions, and fuel consumption. $X_{1}\left(i\right),X_{2}\left(i\right),X_{3}\left(i\right)$ means that both plans 2 and 3 could improve the operating situation, but the improvement ratio has a difference. How to choose a suitable plan for many intersections on road S107 mentioned earlier remains a problem. The EEM was used to calculate the different weights of the six indexes and obtain the final result matrix of plan selection under different traffic volume combinations.

Each plan has six indexes and each index has 45 values. First, we convert the plan-3 result into one matrix. For example, $T_{1}$ is a 45*1 column vector and represents the travel time of plan 1. *T* is a 45*3 matrix as follows:

(10) $$T=\left[\begin{array}{ccc} T_{1}\left(1\right) & T_{2}\left(1\right) & T_{3}\left(1\right)\\ T_{1}\left(2\right) & T_{2}\left(2\right) & T_{3}\left(2\right)\\ \vdots & \vdots & \vdots \\ T_{1}\left(45\right) & T_{2}\left(45\right) & T_{3}\left(45\right) \end{array}\right].$$

The remaining five indexes underwent the same process:

(11) $$\left\{\begin{array}{c} T=[T_{1},T_{2},T_{3}],\\ D=[D_{1},D_{2},D_{3}],\\ S=[S_{1},S_{2},S_{3}],\\ V=[V_{1},V_{2},V_{3}],\\ C=[C_{1},C_{2},C_{3}],\\ F=[F_{1},F_{2},F_{3}], \end{array}\right.$$

where T,D,S,V,C,and F denote the sum of the results of all three plans. $T_{1},T_{2},T_{3}$, etc. denotes the simulation result of each plan.

Second, from each 45*3 matrix, we select the minimum value of each row and output the column number of the minimum value of each row; forty-five column numbers can be obtained and a new matrix named $T_{m}$ generated. $T_{m}$ denotes which plan has the best performance under the same traffic volume combination:

(12) $$T_{m}\left(i\right)=\left[\begin{array}{c} \min T(1,j)\\ \min T(2,j)\\ \vdots \\ \min T(45,j) \end{array}\right],j=1:3.$$

All six matrixes of Equation (11) should be calculated as

(13) $$\left\{\begin{array}{cc} T_{m}\left(i\right)=\min T\left(i,j\right),\\ D_{m}\left(i\right)=\min D\left(i,j\right),\\ S_{m}\left(i\right)=\min S\left(i,j\right), & i=1:45,j=1:3;\\ V_{m}\left(i\right)=\min V\left(i,j\right),\\ C_{m}\left(i\right)=\min C\left(i,j\right),\\ F_{m}\left(i\right)=\min F\left(i,j\right), \end{array}\right.$$

where $T_{m}$ denotes the minimum value of each row of travel time. The remaining five parameters denote the same.

The third step is to calculate the weight of the six indexes and put all six indexes into a single matrix *Y*:

(14) $$Y=\left[\begin{array}{cccccc} T_{m}\left(1,j\right) & D_{m}\left(1,j\right) & S_{m}\left(1,j\right) & V_{m}\left(1,j\right) & C_{m}\left(1,j\right) & F_{m}\left(1,j\right)\\ T_{m}\left(2,j\right) & D_{m}\left(2,j\right) & S_{m}\left(2,j\right) & V_{m}\left(2,j\right) & C_{m}\left(2,j\right) & F_{m}\left(2,j\right)\\ \vdots & \vdots & \vdots & \vdots & \vdots & \vdots \\ T_{m}\left(45,j\right) & D_{m}\left(45,j\right) & S_{m}\left(45,j\right) & V_{m}\left(45,j\right) & C_{m}\left(45,j\right) & F_{m}\left(45,j\right) \end{array}\right],j=1:3.$$

Calculating the weight of six indexes is the fourth step, so that one obtains a scientific method to choose a suitable plan from the three plans.

In information theory, entropy is a measure of uncertainty. The more information there is, the less uncertainty and less entropy there is. According to the characteristics of entropy, we can judge the randomness and disorder degree of a scheme by calculating the entropy value, and we can also judge the dispersion degree of an index by using the entropy value. The greater the dispersion degree of the index, the greater the influence of the index on the comprehensive evaluation. Therefore, the weight of each index can be calculated according to the variation degree of each index by using the tool of information entropy, which provides a basis for the comprehensive evaluation of multiple indexes.

The EEM is an objective weighting method that determines the weight of indicators according to the information provided by the observed values of various indicators. In this article, the data matrix Equation (14) is Y=y(ij)[45×6]. For an index y(j), the greater the gap between the index y(ij), the greater the role of the index in the comprehensive evaluation. If all the index values of an index are equal, the index has no effect in the comprehensive evaluation.

Normalization of indicators: heterogeneous indicators are homogeneous. Since the measurement units of various indicators are not uniform, they should be standardized before the comprehensive indicators are calculated with them; that is, the absolute value of the indicators is converted into a relative value, and $y_{ij}=\left|y_{ij}\right|$, so as to solve the problem of homogenization of different qualitative indicators. Moreover, due to the different meanings represented by positive and negative index values (the higher the positive index value is, the better; the lower the negative index value is, the better), we use different algorithms for data standardization processing for high and low indexes. The specific methods are as follows:

(15) $$y_{ij}=\frac{y_{ij}-\min\left\{y_{ij},...,y_{nj}\right\}}{\max\left\{x_{1j},...,x_{nj}\right\}-min\left\{x_{1j},...,x_{nj}\right\}}.$$

Calculate the weight of index *j* of plan *i*:

(16) $$p_{ij}=\frac{y_{ij}}{\sum_{i=1}^{3}y_{ij}},i=1:45,j=1:6.$$

Calculate the entropy value of the index *j*:

(17) $$e_{j}=-k\sum_{i=1}^{n}p_{ij}ln\left(p_{ij}\right),$$

where k=1/ln(n) and satisfies $e_{j}\geq 0$.

Calculate the entropy redundancy:

(18) $$d_{j}=1-e_{j}.$$

Calculate the weight of each index:

(19) $$p_{ij}=\frac{d_{j}}{\sum_{j=1}^{6}d_{j}}.$$

The weights of the six indexes are shown in Table 6.

The matrix *Y* denotes the plan number corresponding to the optimal value of each index under every traffic volume combination. A matrix A=zeros(45×3) is generated as

(20) $$\sum_{i=1}^{45}\sum_{j=1}^{6}A=\left\{\begin{array}{cc} A(i,1)=w(j) & \mathrm{if}\;\;Y(i,j)=1,\\ A(i,2)=w(j) & \mathrm{if}\;\;Y(i,j)=2,\\ A(i,3)=w(j) & \mathrm{if}\;\;Y(i,j)=3, \end{array}\right.$$

where w(j),j=1:6 represents weight in Table 6. In matrix *Y* (Equation (14)), every row is the different plan numbers of six indexes under every volume combination. For example, the first row of matrix *Y* is

(21) Y[1,.]=[222111].

From Equation (21), it is shown that the first row of matrix *Y* only has the option of plans 2 and 1. Plan 3 did not obtain the best value under this situation. Multiplying the corresponding terms in Equation (21) and Table 6, the weight of plan 2 in Y[1,.] is

(22) w(2)=0.1727+0.1670+0.1262=0.4659.

The weight of plan 1 in Y[1,.] is

(23) w(1)=0.1025+0.2158+0.2158=0.5341.

The first row of matrix *A* is

(24) A[1,.]=[0.53410.46590].

In matrix *A*, each column represents plans 1, 2, and 3. The results in every row of matrix *A* denote the different weights of plans under corresponding volume combinations. Because 0.5341>0.4659, plan 1 is the final choice of the first row in matrix *Y*, which is the first volume combination, i.e., arterial volume 686 veh/h and collector volume 100 veh/h.

Based on Equations (20)–(24), we generate a matrix *T* to save the final selected plan number of each row:

(25) $$T(i)=\left\{\max A[i,.]\right\}\left(j\right).$$

**Figure 9:**
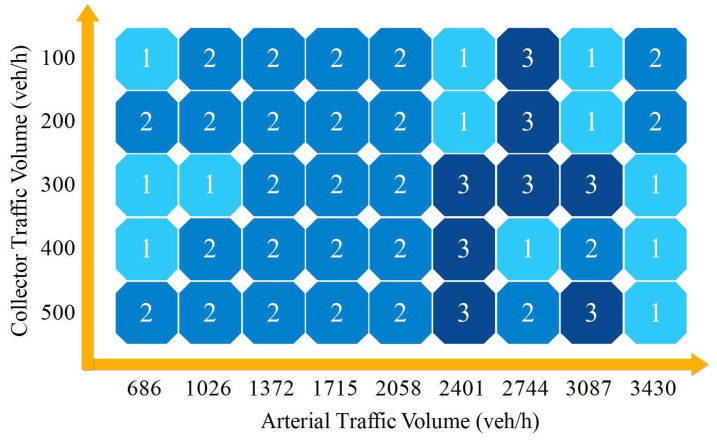
Selection matrix for different plans. 1, 2, and 3 represent plans 1, 2 and 3, respectively. Different volume combinations correspond to different plans.

Matrix *T* contains 45 plan numbers, which are the final choices of all volume combinations. Transposing matrix *T* into a 9×5 matrix, we obtain the results plotted in Figure 9.

Figure 9 shows the final plan choice under all 45 volume combinations. Plan 1 appeared on both sides when the arterial volume ranged from 686–1026 veh/h to 2401–3430 veh/h on the horizontal axis. When arterial volumes were 686 and 3430 veh/h, plan 1 occupied three blocks; when the arterial volumes were 2401 and 3087 veh/h, plan 1 occupied two blocks; and when the arterial volumes were 1026 and 2744 veh/h, plan 1 only showed in one block. From the vertical axis (the collector volume), plan 1 appeared three times when the collector volumes were 100, 300, and 400 veh/h, and twice and once when the collector volumes were 200 and 500 veh/h, respectively.

Plan 2 in Figure 9 was distributed mainly in left and central parts. Plans 2 and 1 were interspersed in the columns of arterial volumes of 686 and 1026 veh/h. All 15 blocks between 1372 and 2058 veh/h shown in blue represent that plan 2 is the best choice in this large range. Plan 2 was distributed sporadically when arterial volumes were larger than 2401 veh/h. Plan 2 could be mainly used under 2058 veh/h, and especially between 1372–2058 veh/h.

Plan 3 in Figure 9 appeared centrally and only occupied eight blocks in the entire scale. When arterial volumes were less than 2058 veh/h, plans 2 and 1 performed better than plan 3. Plan 3, signal control, could be useful when both arterial and collector volumes are large.

Collector street volume also has some influence on plan performance, especially arterial volumes larger than 2401 veh/h. This means that the plan choice was based mainly on arterial street volume, but, when arterial volumes were larger than 2401 veh/h, the plan choice depends on collector street volume.

**Figure 10:**
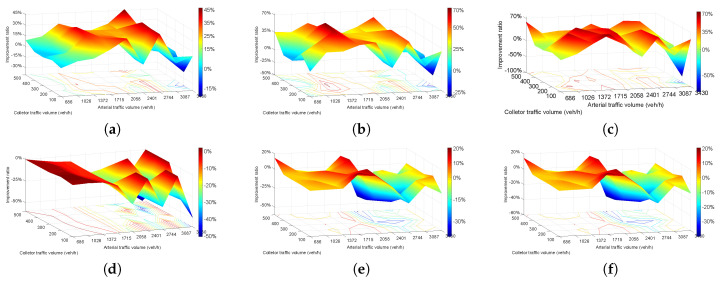
Operating performance of Figure 9 matrix. (**a**) travel time; (**b**) delay; (**c**) number of stops; (**d**) vehicle number; (**e**) CO emissions; (**f**) fuel consumption.

Based on Figure 9, we selected the corresponding index values and compared the improvement ratio with the present situation (plan 1), and the results are shown in Figure 10.

Figure 10a denotes travel time based on Figure 9. Travel time was reduced obviously over the entire range, except for an arterial volume of 3430 veh/h. In the range of arterial volumes 1372–2744 veh/h, travel time was generally reduced by 25% and reached a peak value of 45% at 2744 veh/h. Compared to Figure 7a and Figure 8a, Figure 10a has two peak values that combine two advanced parts.

Figure 10b shows delay based on Figure 9. This figure avoids the negative change of Figure 8a when arterial volume was less than 1372 veh/h, from almost −300% increased to −25% at most, which is a great improvement.

Number of stops is shown in Figure 10c, and Figure 10b avoided the negative change of Figure 7c and Figure 8c. The majority situations of the entire scale improved, except for an arterial volume of 3430 veh/h and a collector volume of 200 veh/h.

Number of vehicles has a strong decreasing trend over the entire range in Figure 7d and Figure 8d. With the EEM calculation, the strong trend has a more significant change than before. The original reduced area has four peak values, which is significant progress.

Figure 10e,f denote CO emissions and fuel consumption, and they exhibit the same trend. Both indexes were reduced by up to 20% when arterial volumes was less than 2058 veh/h, and then increased after that. The increase of CO emissions and fuel consumption could not be avoided because of the large volume of vehicles. When the main indexes of travel time and delay increased, CO emissions and fuel consumption also increased.

In general, with the EEM calculation, the final plan choice could make the traffic operation smoother and maximize traffic capacity, thus avoiding the single reconstruction plan disadvantages and showing an easy number matrix for traffic management departments to choose a suitable plan for different intersections. With the EEM, travel time, delay and number of stops could reduce obviously 70% at most, CO emissions and fuel consumption are also reduced up to 20%, which is a great improvement with traffic volume reduced gently.

The length of the simulation road in VISSIM is 630 m. The total length of the real road in Figure 2 is 84.3 km with 87 uncontrolled T-intersections. If all 87 T-intersections will be reconstructed by EEM, the optimized segment length will reach 630 × 87 = 54,810 m = 54.8 km, which accounts for 65% of the whole road. The improvement of six indexes for one single T-intersection with EEM is shown in Figure 9. For the whole road, the maximum optimization of six indexes, travel time, delay, number of stops, number of vehicles, CO emissions and fuel consumption could reach 29%, 45%, 45%, 26%, 13% and 13%, respectively. This degree of optimization is significant, and, even if the optimization method in this paper only works in half of the cases, the reduction is still huge. This is a great optimization result. In comparison, the automatic start-stop technology popularized by vehicles in recent years can save fuel by 3–10% [82,83]. The research method in this paper can achieve the same fuel saving level as automatic start-stop, which is very significant for the whole society to save energy and reduce emissions.

### Verifying the Validity of the EEM

The collected data could be used to verify the validity of the EEM. The collected real traffic data are presented in Table 4, showing that the arterial volume is 2040 veh/h in two directions and the collector street volume is 200 veh/h. From the matrix in Figure 9, the EEM result shows that plan 2 is the best choice under this volume combination. The three plans are compared below.

**Figure 11:**
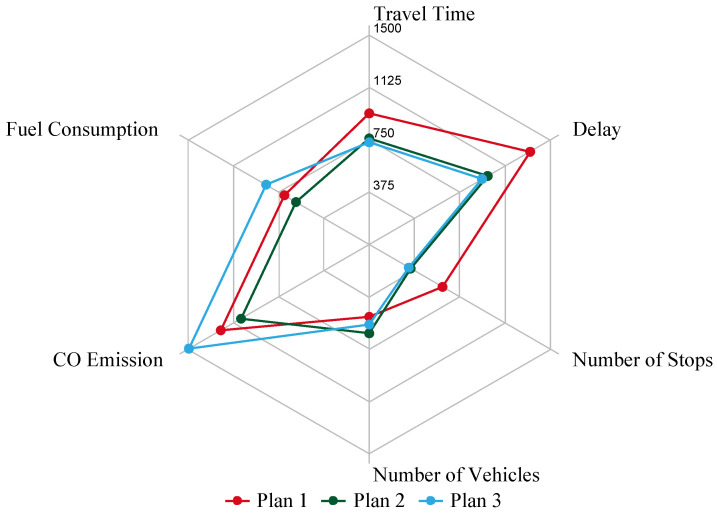
Performance of plans 1, 2, and 3 with collected data in Table 4. Because delay, number of stops, and fuel consumption values were very small, the values were enlarged 20 times.

The comparison of the three plans with the collected data from Table 4 are shown in Figure 11. Plans 2 and 3 had an obviously better performance than plan 1 on travel time, delay, and number of stops. In addition, plans 2 and 3 were very similar and without obvious differences in these three indexes. For number of vehicles, plan 2 > plan 3 > plan 1. For CO emissions and fuel consumption, they had that same relation, namely that plan 3 > plan 1 > plan 2. Therefore, it is very easy to find that plan 2 was the best option of the three plans with collected data and exhibited the same result shown in Figure 9, which verifies that the plan choice made using the EEM is reliable.

## 5. Conclusions

China is in the rapid development stage of urbanization, with the rapid increase of urban population and continuous growth of vehicles, traffic congestion and air pollution becoming increasingly serious. The main measurement is vehicle restriction policy, forbidden nonlocal vehicles and restricted use of local vehicles, for the majority of Chinese cities to reduce traffic congestion and emissions. The administrative rule is simple and crude, which will have a negative impact on citizens’ travel and regional economic development. Tapping the potential of the existing road network, especially the low-cost reconstruction method of existing roads, is essential to improve traffic condition, and reduce congestion and air pollution.

At present, having no median strip or waiting area at intersections has a strong negative effect on, and poses a safety hazard for, passing traffic flows. In this study, two improved schemes, channelization and signalization, were both compared with the present situation. A VISSIM simulation model was developed and calibrated to evaluate the present features, and the channelization and signalization were also evaluated for comparison. Six indexes—travel time, delay, number of stops, number of vehicles, CO emissions, and fuel consumption—were used to evaluate the three plans.

The results show that the three plans have the best performance under different situations on T-intersections. Plan 1, the present situation, could be used for low arterial volumes, e.g., 686 veh/h. Plan 2, channelization, could be mainly used when arterial volumes are between 1372 and 2058 veh/h. Plan 3, signalization, only appeared in the area when arterial volumes were larger than 2401 veh/h, but all three plans were mixed in this section. All three plans could not solve the traffic problem when the arterial volume was 3430 veh/h, which corresponds to service level E in [59], as all six indexes were seriously deteriorated.

The calculation in this paper is only for one intersection, if a city as large as Xi’an could use this method in the whole city. It will be significant to reduce the driving interference of vehicles at intersections, acceleration and deceleration distance, number of stops and exhaust emissions. The EEM method in this study is simple and clear for engineers to learn and apply. These findings can be utilized as a guideline for traffic police departments and road designers to determine when, where, and how the different plans should be used, and which intersection has the priority in reconstruction. This is also an attempt to use a technical method instead of administrative vehicle restriction policy for reducing traffic congestion and emissions. Before the method is used in the future, some issues should be studied first. First, an exclusive right-turn lane can also be designed for the two right-turning flows to isolate the influence of the other flows. Second, this study only covers a three-direction T-junction, so how the EEM should be used and implemented for a four-direction junction also needs further study. The authors recommend that future studies focus on these issues.

## Figures and Tables

**Table 1:** Several restriction policies in various Chinese cities.

Cities	Monday	Tuesday	Wednesday	Thursday	Friday	Note
Xi’an/Chengdu	1, 6	2, 7	3, 8	4, 9	5, 0	07:00–20:00
Beijing	0, 5	1, 6	2, 7	3,8	4, 9	The number changes every 3 months
Shanghai	Vehicles from other provinces all forbidden on weekdays					
Guangzhou	Drive consecutively for 4 days at most, stop driving for 4 days consecutively					

The restriction depends on the last number of the plate. The restriction policy in Xi’an and Chengdu are the most common rules in China. Beijing has a similar policy, but the restriction day changes every three months—for example, 0 and 5 are restricted on Monday from 20190408–20190707 while changes to Tuesday from 20190708 to 20191006. Nonlocal vehicles are forbidden in Shanghai 24 h on weekdays. The policy in Guangzhou is the most complex, drive 1, 2, 3 or 4 consecutive days at most, then stop driving for four consecutive days, then you can drive for 1, 2, 3 or 4 days, regardless of whether it is a weekday or a weekend.

**Table 2:** Geometric parameters of ELTL when design speed is 80 km/h.

Item	Description
$L_{AB}$&$L_{AE}$	Symmetry design, 12.15 m length, radius is 22.5 m. Entrance and/or exit of ELTL for flow i=3,5 to turn
$L_{BC}$	190 m. Acceleration length for flow i=5 to accelerate to design speed
$L_{CD}$	132 m. Length for seeking a headway for flow i=5 and merging into flow i=1
$L_{EF}$	27 m. Wait area length in case of flow i=3 needing to wait to turn
$L_{FG}$	90 m. Deceleration length for flow i=3 from design speed to stop
$L_{GH}$	50 m. Length of diversion to separate flow i=1 and flow i=3

**Table 3:** Number of vehicles in the collection.

Time	Friday	Saturday
Morning	2031	1944
Middle noon	1530	1836
Evening	2195	2240

**Table 4:** Vehicle information collected during investigation.

Item	East to West		West to East		Collector Street	
Flow	i=1	i=3	i=2	i=4	i=5	i=6
Car	920	40	898	80	108	92
Truck/Bus	50	0	52	0	0	0
Average speed (km/h)	45.5	16.7	37.3	11.5	18.4	8.5
Max. speed (km/h)	81.4	23.5	91.4	25.7	28.5	12.3
Min. speed (km/h)	25.2	0	0	0	0	0

Minimum speed is 0 km/h, meaning some vehicles stop and wait to move, including TVs waiting to cross the street and SVs waiting to pass through.

**Table 5:** VISSIM simulation calibration results.

Flow	i = 1	i = 2	i = 3	i = 4	i = 5	i = 6
Investigated capacity (veh/h)	970	950	40	80	108	92
Simulated capacity (veh/h)	936	864	50	90	90	108
Individual MAPE (%)	3.5	9.0	25.0	12.5	16.7	17.4
MAPE (%)	4.3					

**Table 6:** Weights of six indexes.

Index	*T*	*D*	*S*	*V*	*C*	*F*	Summation
Weight	0.1727	0.1670	0.1262	0.1025	0.2158	0.2158	1.0000
